# Antiviral attributes of bee venom as a possible therapeutic approach against SARS-CoV-2 infection

**DOI:** 10.2217/fvl-2023-0127

**Published:** 2023-11-07

**Authors:** Soumik Goswami, Jayita Pal Chowdhury

**Affiliations:** ^1^Department of Zoology, Sunbeam Women's College, Varuna, Varanasi, 221002, India; ^2^Department of Zoology, Banaras Hindu University, Varanasi, 221005, India

**Keywords:** bee venom, cell lysis, inflammation, melittin, SARS-CoV-2

## Abstract

The unprecedented scale of the SARS-CoV-2 pandemic has driven considerable investigation into novel antiviral treatments since effective vaccination strategies cannot completely eradicate the virus. Apitherapy describes the medicinal use of bee venom, which may be an effective treatment against SARS-CoV-2 infection. Bee venom contains chemicals that are antimicrobial and stimulate the immune system to counteract viral load. The present review focuses on the use of bee venom as a possible treatment for COVID-19 and reviews studies on the pharmacodynamics of bee venom.

The competitive co-evolution of hosts and pathogens has always proved to be a significant driving force for scientific research. SARS-CoV-2, the virus causing COVID-19, emerged in the Wuhan province in China in 2019 [1,2]. This novel virus was entirely new and by April 2020, the disease was escalated to a global pandemic by the WHO. Initial mortality rates of 5% accelerated to 6.7% within a week [3,4]. COVID-19 also seriously affected other areas of human welfare, like economic status, social status and mental well-being. SARS-CoV-2 is a virus belonging to the coronavirus super family characterized by the presence of spike proteins that facilitate the viral entry into host cells via the ACE-2 receptor, a predominant feature of host cells [5,6]. ACE-2 enhances the replication of the viral particle and positively influences the invasion of the host cell [7]. The pathogenesis of the SARS-CoV-2 infection triggers several responses within the body like the induction of cytokine storms and hyper-inflammation, and often culminates in multi-organ failure [8,9]. Cardiovascular problems are also attributed to COVID-19 patients because ACE-2 interacts with the renin angiotensin aldosterone system (RAAS) predominant in the nephrons and also interacts with the enzymes of the cardiovascular system [10,11]. SARS-CoV-2 has a dangerous ability to evade the immune system. The subsequent stages of infection involve the increase of viral propagation, generation of an active immune response, and the spread of the virus to the lower respiratory system leading to gradual infections of the digestive and cardiovascular systems, which are cumulatively attributed to the second stage of infection [12]. The clinical manifestations worsen in the third stage of infection characterized by symptoms like hypoxia, infiltration of the entire respiratory system, and ultimately a state of acute respiratory distress syndrome (ARDS), which often proves to be fatal [11]. Initial approaches of utilizing polyclonal antibodies generated in a COVID-19-recovered patient proved to be beneficial but it had its own detriments, due to allergic reactions [13]. Chloroquine and its derivatives were also used to treat patients exhibiting mild symptoms [8]. Other drugs tested include the anti-ulcer drug famotidine, which initially proved effective [14], and antiviral drugs like Remdesivir [15], which was effective at relieving severe symptoms and preventing fatalities, and lopinavir/ritonavir drug, which is a predominant anti-HIV drug [16–18]. Hence, the necessity for a therapeutic with minimal side effects and maximum antiviral properties has become important to address. Venom from honey bees is a promising agent that can be highly significant in relieving the severe effects of SARS-CoV-2. It has been experimentally established that bee venom has the ability to lower the RAAS parameters and lower serum Angiotensin levels in laboratory subjects [14]. Hence, this establishes a connection of bee venom with the ACE-2 pathway which can be targeted for preventing the successful invasion of the host cells by the virus.

## Bee venom: properties & variability in composition among bee variants

**Figure 1.**
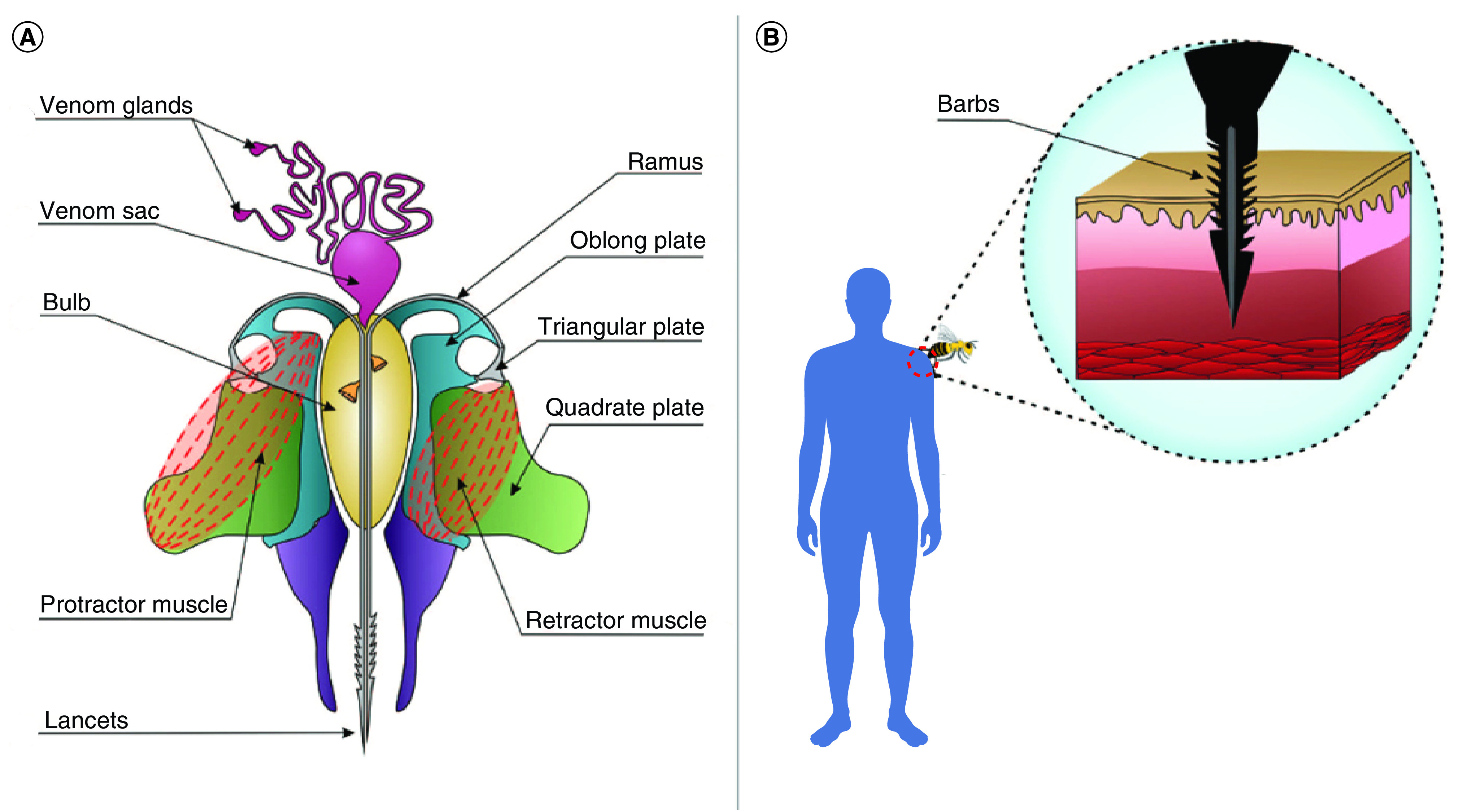
Stinging apparatus and mechanism of stinging in honey bees. **(A)** Stinging apparatus of a honey bee. **(B)** The process of stinging by a honey bee using the barbules of the sting apparatus. Reproduced from Pucca MB, Cerni FA, Oliveira IS *et al.* with permission from [44].

Apiculture or the rearing of honey bees is a fairly common practice in almost all countries, including India. It provides a cheap, fast and reliable way of utilizing the products of honey bees, which include propolis, royal jellies, bee wax and honey. Bee venom (BV) is another very important component of honey bees. The venom in bees is stored in venom sacs located on the posterior side of the abdomen and it is released via the sting apparatus which punctures the skin as a bee stings (Figure 1). The release of venom during the stinging is a defensive behavior adopted by bees upon encountering threats.

The venom of honey bees is a complex cocktail of several biomolecules and thus the functionality cannot be attributed to any single component. Bee venom constituents include different enzymes like phospholipaseA2 (PLA2), phospholipase B, hyaluronidases, acid phosphatases, acid phosphodiesterases, a-D-glucosidases and lysophospholipases. The non-enzymatic peptides in bee venom include melittin, apamin, mast cell degranulating peptide, secapine, pamine, minimine, procamine A, B, protease inhibitor, tertiapine, cardiopep and adolapin [19–22]. Bee venom also contains different amino acids like g-aminobutyric acid and α-amino acids. The presence of amines has also been reported in bee venom and this includes dopamine, histamine, norepinephrine and neurotransmitters [23,24].

The deleterious effects of the chemical constituents of bee venom include prominent inflammation, allergic reactions to protease inhibitors, anaphylactic responses and death [25]. However, these damaging effects are manifested at extremely high doses. Allergic responses toward bee venom are mainly attributed to the presence of the peptides which are antigenic to humans [26]. Manifestations of these allergic reactions are observed in different physiological systems including the respiratory system, gastrointestinal system, cardiovascular system and skin. The sting of honey bees can also induce hypersensitivities culminating in anaphylactic shock, which can also lead to cerebral or myocardial ischemia [27]. However, there are several reports depicting the anti-inflammatory, anti-nociceptive, antioxidant and anti-apoptotic properties of bee venom [28–30].

**Figure 2.**
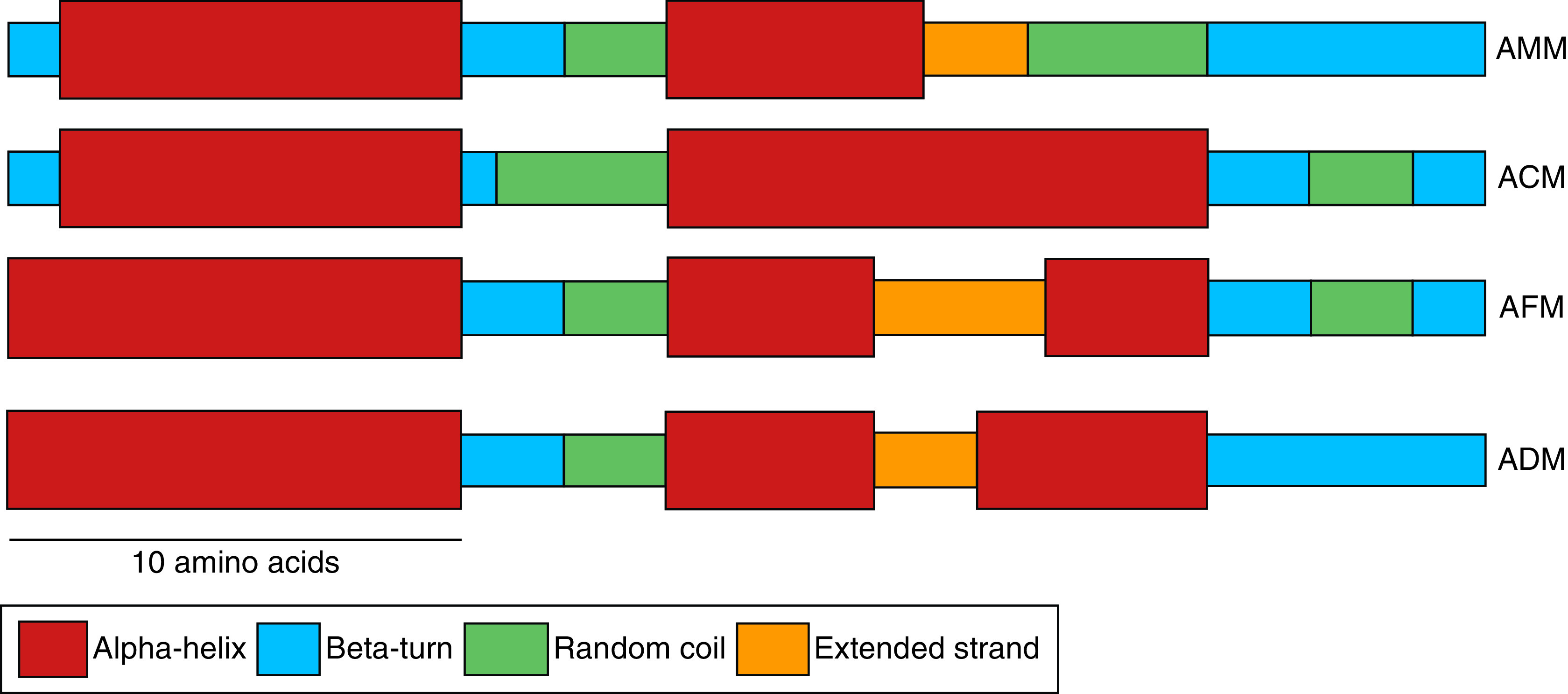
Structure of melittin from four different species of honey bees based on the secondary structural analysis via self optimized prediction method from alignment. The structural analyses of melittin showed a predominance of alpha-helices (red), followed by beta-turns (orange), random coils (green), and extended strands (blue). The scale bar represents the lengths of 10 amino acids. Adapted from Park D, Jung JW, Lee MO, *et al.* with permission from [32].

Since there is significant species diversity among honey bees, the composition of the venom also varies accordingly in different species. These differences might be an adaptive response according to the habitats the bees reside in. The concentration and structural variability of melittin is exhibited in different species of honey bees (Figure 2). Melittin concentrations in *A. dorsata, A. mellifera, A. florea* and *A. cerana* were reported to be 95.8 ± 3.2%, 76.5 ± 1.9%, 66.3 ± 8.6% and 56.8 ± 1.8%, respectively [31]. The body size of the honey bees positively correlates with the size of the venom sacs and the venom apparatus. Hence, *A. dorsata* being the largest of the different species has the largest venom gland and venom sac, followed by *A. cerana, A. mellifera and A. florea.* Besides, it has also been reported that the concentrations of constituent lipid, protein, carbohydrate and alkaline phosphatase are usually the most in *A. cerana*, followed by *A. mellifera and A. florea* [33]. Experimental evidence suggests that the crude venom and melittin taken from *A. cerana* exhibited the most significant antimicrobial activity against Gram-positive bacteria compared with Gram-negative bacteria and fungus, followed by *A. mellifera, A. florea and A. dorsata* [33]. It has also been reported that crude bee venom shows more antimicrobial activity than synthetically procured melittin alone. Hence, although melittin is the most active compound the other chemical components of the bee venom cumulatively exhibit a combine and synchronized anti-microbial activity.

## Medical uses of bee venom

A properly directed low concentration and diluted BV has been shown to trigger a wide range of anti-inflammatory responses [34–36]. Hence, such doses are extensively used in medical conditions of diabetes, asthma, skin diseases, rheumatoid arthritis, obesity and cardiovascular diseases [37]. Low doses of BV suppress the pro-inflammatory cytokines like IL-6, IL-8, IFN-γ and TNF-α, which depicts the potential of BV to ameliorate conditions of severe inflammation which is a predominant feature of SARS-CoV-2 pathogenesis [37]. The ability of the BV to act as an anti-inflammatory molecule is partly by the suppression of the cytokines but scientific reports also suggest that that BV can regulate the inflammatory biosignaling involving NF-κB, extracellular signal-regulated kinases (ERK1/2) and protein kinase Akt in porphyromonas gingivalis lipopolysaccharide (PgLPS) administered human keratinocytes [38]. Immune reactions elicited by bee venom are toxic at high doses. However, controlled dilutions of bee venom have been reported to be positive modulator of immune reactions. Therefore, these controlled hypersensitivity reactions are beneficial for establishing a strong defense system against antigens. BV can also be administered as a pain-killer since it inhibits cyclooxygenase activity and attenuates the prostaglandin synthetase system at controlled dose concentrations [39–41]. At desired dilutions, BV can also induce anti-nociceptive effects by targeting the α-adrenergic receptor [42,43].

The Western honey bee's (*Apis mellifera*) venom has been used since the ancient Egyptian era (4000 BC) [45]. The beneficial effects of bee venom have also been reported for several diseases, including arthritis, rheumatism, pain, cancerous tumors and skin diseases [46]. The venom of Western honey bee was found to show multiple beneficial effects, comprising anti-inflammatory [47], antimicrobial [45,48–50] and antioxidant activities [47,51]. Honey bee venom contains different chemical substances like melittin, apamin, adolapin and mast cell degranulating peptides; it also contains enzymes like phospholipase A2 (PLA2) and hyaluronidase; biologically active amines like histamine and epinephrine [45]. Melittin is the predominant peptide found in bee venom and the total concentration of the peptide is 40–50% of the venom's dry weight [46]. Melittin is positively charged and is amphipathic in nature, consisting of 26 amino acid residues. The initial 20 amino acids are hydrophobic and are located toward the amino-terminal of the peptide [52]. The remaining six amino acids are hydrophilic and are present toward the carboxy-terminal end of the peptide. Melittin is water-soluble and upon aqueous solution of melittin consists of the peptide existing as a monomer or a tetramer [53]. Of all constituents of bee venom, melittin is the most potent compound exhibiting antimicrobial and cytotoxic properties [54].

The medical use of BV was established during the times of Hippocrates when the toxin was used to treat conditions of arthritis and joint pain [55]. There has also been extensive use of BV in treating neurological problems like multiple sclerosis, Alzheimer's disease and Parkinson's disease [56]. The administration of bee venom can be either directly from the sting of bees or via injectable routes. The sting of honey bees can, on the contrary, evoke several effects on the body, like allergic reactions such as anaphylactic shock [57] and in severe cases; the stress response of the body against the toxins may even prove to be fatal [58].

## Melittin as the target molecule against SARS-CoV-2

The administration of BV has also been associated with the differentiation of regulatory T cells [59]. However, these responses of the body might prove to be a positive aspect if utilized in a proper way. These allergic reactions might be the answer to SARS-CoV-2 [60,61] since the regulatory T cells have a role in the control of the virus [62]. The prime event of an allergic reaction is the generation of IgE antibodies and this is also seen in the case of BV exposure [61], but the induction of IgE production might also be helpful against the virus because IgEs have a positive response against a wide plethora of antigens [63]. It has also been experimentally observed that BV can also be used as an adjuvant with Toll-like receptor ligand [64], wherein it was effective in enhancing the population of foxP3 expressing cells and augmenting the concentration of circulatory regulatory T cells [65].

SARS-CoV-2 belongs to the β genus of the family of coronaviruses which has a protective protein covering enclosing the viral genome. There are four main types of structural proteins in SARS-CoV-2, which are categorized as membrane, spike, nucleocapsid and envelope protein. The membrane of the virus contains 3–4 glycoproteins [66], which are the most abundant protein forms and these span the membrane bilayer thrice in such a way that the COOH terminal remains within the virus while the NH2 terminal remains outside the virus. The spike protein of the SARS-CoV-2 facilitates the attachment of the virus with the host cells. These are structurally Type I membrane glycoproteins and form people that are involved in antibody interactions [66]. SARS-CoV-2 has been shown to produce fewer forms which are non-infectious in the presence of tunicamycin [67]. Therefore, this is the exact point of entry of the virus which needs to be targeted in order to prevent it from entering into the subsequent stages of infection. Melittin is the water-soluble predominant compound in BV and hence the administration via injection becomes relatively less complex. Moreover, melittin has been known to exhibit cell lysis properties by inflicting pore formation on the membrane of the cell [68]. There has been extensive research showing the therapeutic potential of melittin as a possible anti-arthritic, anti-neoplastic and anti-inflammatory agent [69]. The ability of the compound to inflict cell lysis has also been effectively utilized against microbes like different bacteria & and viruses, of which notable effects have been observed in the case of Herpes simplex virus, H1N1 Influenza A virus, etc. [70]. The other attribute of melittin which still further promises to be beneficial against viruses is its ability to stimulate the production of type I interferons [71], which is the body's natural anti-viral chemical. Melittin has been experimentally proven to induce pore formation in the outer coat of the Human Immunodeficiency virus (HIV) [67]. Further studies have also shown that targeted delivery using melittin-loaded nano-particles intravenously has successfully killed pancreatic lesions in K14-HPV16 mice with squamous dysplasia and carcinoma containing human papillomavirus (HPV) transgenic elements [72,73]. Melittin might be the answer that we have been trying to search for the past year to achieve a potential therapeutic strategy toward SARS-CoV-2. Following primary research and depending upon the results, there can be further extensive research wherein the targeted delivery systems can be employed to directly affect the virus-infected cells without harming the neighboring cells.

## Conclusion

**Figure 3.**
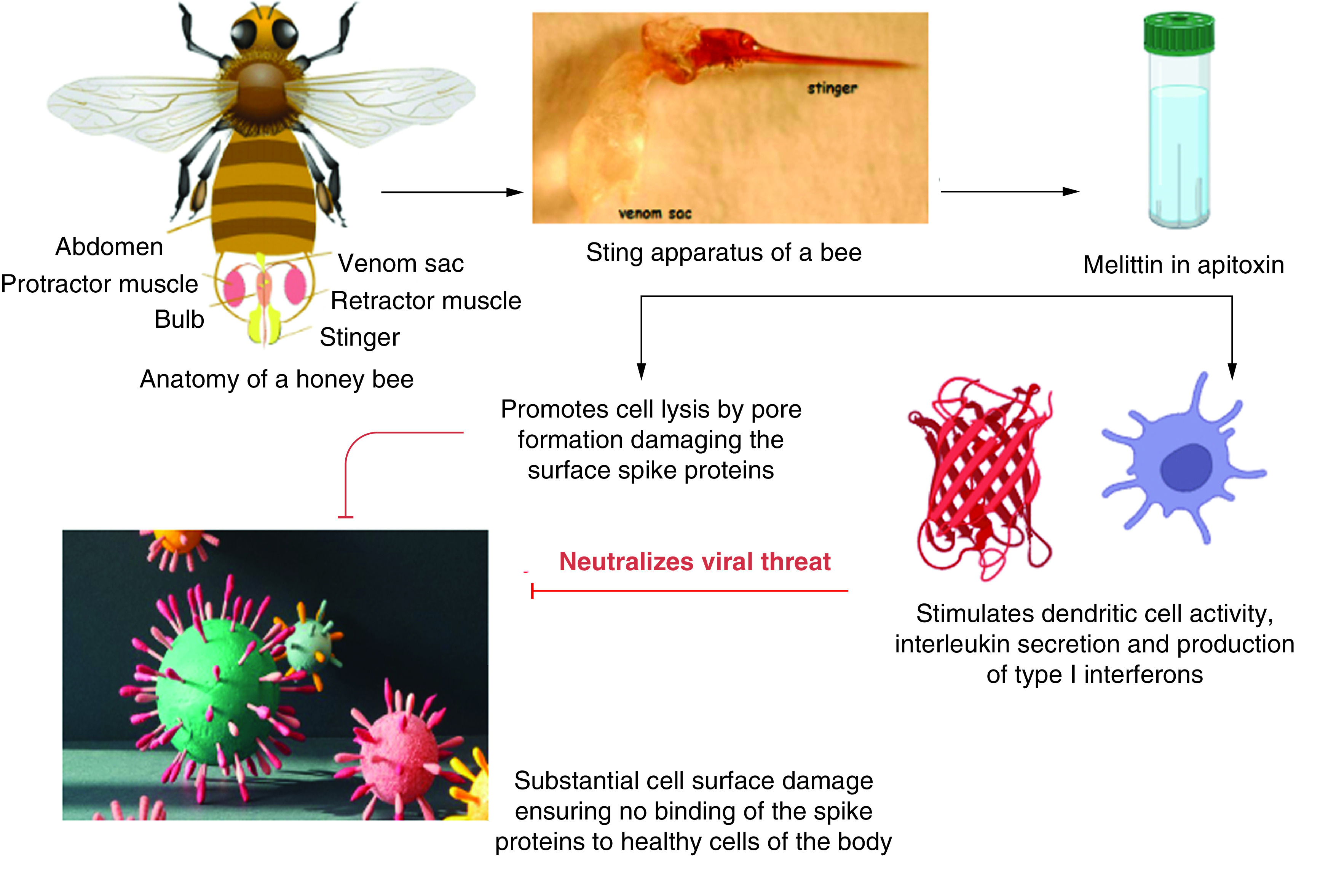
A summary of the hypothesis of this manuscript.

Bee venom might be an interesting form of medical intervention to prevent the severe form of COVID-19. The direct association of BV components with ACE-2 substantially points to the direct connection for utilizing BV against SARS-CoV-2, which initiates its cellular entry via the ACE-2 receptor (Figure 3). The present situation has seen the trials of several drugs to counteract the harmful effects of the virus and we can surely not expect a magical drug to emerge suddenly that will potentially neutralize the virus. However, combinatorial therapy might be a good approach to follow as it increases the ability of drugs to neutralize pathogens in an effective way. The anti-viral, interferon-inducing and pore-forming ability of melittin and other compounds in BV will definitely be effective against melting SARS-CoV-2. Although it is too early to nominate BV as the drug against SARS-CoV-2, it can surely be presumed that BV might be a potential complementary medicine against SARS-CoV-2 and its further aggravation into severe forms of COVID-19 and long-term.

## Future perspectives of bee venom in complementary medicine

Controlled and low doses of bee venom have shown significant anti-inflammatory responses which have been utilized as a speculative therapeutic approach in diabetes, rheumatoid arthritis (RA), cardiovascular diseases, obesity, asthma, skin diseases and neurodegenerative disorders like Alzheimer's disease, Parkinson's disease and amyotrophic lateral sclerosis [74,75]. This anti-inflammatory property of bee venom is by the suppression of pro-inflammatory cytokines like IL-6, IL-8 and TNF-alpha, involving an NFκB-mediated pathway. The other important attribute of bee venom is the attenuation of tumor progression and this property is primarily exhibited by the binding of phospholipase A2 to phosphatidyl-inositol, 3,4 biphosphate, or dendritic cells (DCs) [76]. Phospholipase A2 present in bee venom is a membrane-binding protein and attaches antigens to the plasma membrane of human DCs *in vivo*. This presentation of antigens stimulates a T-cell (CD8+ve) mediated immune response which promises to manifest as a possible anti-viral and anti-tumor vaccine model in the future. Vaccines from BV and DCs (cell-based antiviral/antitumor vaccines) are used for immunization against viruses such as cytomegalovirus and for suppression of tumors [77,78]. Aluminum hydroxide is presently used as an adjuvant for SARS-CoV-2 infections for immune therapies and there are significant speculations about the use of bee venom as a potential adjuvant in immune therapies adopted against COVID-19. PLA2 has been associated with a level of success against SARS-CoV-2 infections [79,80]. Combinatorial or complementary medicines certainly prove to be beneficial in such aspects of novel viruses and initial studies with the bee venom chemicals have provided promising results. However, the exact cellular signaling pathway involved in the amelioration of the viral damages needs to be elucidated further. Janus kinase (JAK) signaling has been an area of focus for COVID-19-related physiological anomalies. Therefore, the exact relationship between bee venom proteins, JAK and subsequent downstream signaling molecules might unravel a new area of pharmacodynamics for the venom constituents.

Melittin-loaded nano-particle formulations might be the future approach to neutralize SARS-CoV-2 and prevent it from culminating into COVID-19. There also can be gel formulations in the form of nasal sprays containing melittin-loaded nano-formulations which might stop the spread of the virus from the nasopharyngeal region. An interesting survey report of 5115 beekeepers in the Hubei province of China, among which 723 beekeepers were directly from Wuhan, which was the epicenter of the COVID-19 pandemic symptoms, showed no COVID-19 symptoms [81]. A similar study was also conducted among five apitherapists residing in Wuhan and 121 patients who regularly undergo treatments at their respective clinics. It was again observed that two out of the five apitherapists were exposed to suspected SARS-CoV-2 infected people and three out of the five were definitely exposed to confirmed COVID-19 patients. Surprisingly, all the five apitherapists did not develop any symptoms of the disease and none of the 121 patients developed any symptoms either [81]. Therefore, this study substantially depicts the development of a tolerance against SARS-CoV-2 in people exposed to BV. This data clearly shows the importance of BV as a potential therapeutic approach against the anomalies exhibited by SARS-CoV-2 virus infection.

**Executive summary**

- Sudden outbreak of the novel SARS-CoV-2 virus and its severe consequences on human health.
- Medications and vaccines administered to date are not completely beneficial against the virus.
- There is a need for complementary medicine that can be obtained by utilizing the antimicrobial properties of the constituents of bee venom.

**Bee venom: properties & variability in composition among bee variants**

- Bee venom is a complex cocktail of peptides, enzymes, amines, etc.
- Melittin is the predominant peptide that has shown antimicrobial effects in different experimental setups.
- Bee venom definitely exerts severe allergic responses and inflammation but only at high doses.
- The venom content and its chemistry differ among different species of honey bees.
- Melittin is the predominant peptide found in the venom of almost all species of honey bees.

**Medical uses of bee venom**

- The possibility of using bee venom as complementary medicine against COVID-19.
- Low and controlled dosing of bee venom or its components can be of significant medical importance against SARS-CoV-2.
- Melittin has the ability to induce pore formation in the cell membrane and also stimulates the immune system by triggering the release of interferons.
- These properties of melittin have been utilized against Herpes virus, H1NI influenza, etc., and can also be of immense significance against SARS-CoV-2.

**Melittin as the target molecule against SARS-CoV-2**

- Melittin is the water-soluble predominant compound in BV and hence the administration via injection becomes relatively less complex.
- Melittin has been known to exhibit cell lysis properties by inflicting pore formation on the membrane of the cell.
- The ability of the compound to inflict cell lysis has also been effectively utilized against Herpes simplex virus, H1N1 Influenza A virus.
- Melittin-loaded nano-particles intravenously has successfully killed pancreatic lesions in K14-HPV16 mice with squamous dysplasia and carcinoma containing human papillomavirus (HPV) transgenic elements.
- Melittin might be the answer that we have been trying to search for the past year to achieve a potential therapeutic strategy toward SARS-CoV-2.

**Future perspectives of bee venom in complementary medicine**

- Melittin has proven to be beneficial in real-life case histories wherein beekeepers involved in apiculture did not suffer the consequences of SARS-CoV-2 in China. Similar results were also obtained among apitherapists.
- Nasal formulations containing nano-formulations loaded with melittin might be a futuristic strategy to employ against restricting the SARS-CoV-2 to the nasal passage only.
- Apart from melittin alone, phospholipase A2 has also been scientifically proven to induce T-cell-mediated immune response by targeting dendritic cells for antigenic processing and presentation.
- Bee venom-derived anti-viral or anti-microbial vaccines are the future therapeutic strategy against most viral antigens.
- Melittin and phospholipase A2 might be the replacement for aluminum hydroxide as an adjuvant utilized in immune therapy in COVID-19.
